# A qualitative study of the benefits and challenges of different models of extra care housing for residents living with dementia

**DOI:** 10.1177/14713012241249794

**Published:** 2024-05-03

**Authors:** Rebecca Oatley, Teresa Atkinson

**Affiliations:** School of Social Sciences, 2112 Cardiff University, UK; Association for Dementia Studies, 8709 University of Worcester, UK

**Keywords:** extra care housing, housing with care, dementia, independent living, specialist

## Abstract

Extra care housing (ECH) is a type of housing with care and support designed to enable older people to age in place. Approximately one fifth of residents living in ECH are living with dementia and yet, there remains gaps as to how best to support people to live well with dementia in the context. ECH stock across the United Kingdom (UK) includes a diverse range of options that can be grouped into integrated, specialist and separated accommodation. Integrated models involve residents with dementia living alongside residents without dementia. Specialist ECH offer accommodation exclusively for people living with dementia. Separated models offer a separate area for residents with dementia within a larger, integrated site. How these different models work for residents living with dementia is little known and has remained a significant gap in knowledge that impairs both professionals and people living with dementia when choosing housing and care. This paper reports on findings from a large study of residents living with dementia in ECH. The focus is on the potential benefits and challenges of different models of provision. Data were generated from interviews with 100 participants (residents, family members, staff, and adult social care professionals) at eight case study sites across England. Findings demonstrated that there are potential benefits and challenges within each model, but the limited diversity of stock limits choice. Multiple variables beyond the model of provision affect the lived experience, meaning that there is no universal model of optimal support. Rather, the approach and resources of each site is more important than the model of provision. Suggestions for future research directions are considered.

## Background

Increasing numbers of people living with dementia is the biggest challenge faced by adult social care (United Kingdom (UK) Government, 2015). Housing is inextricably linked with care and support provision (Twyford & Porteus, 2021) and suitable housing can sustain independent living, community connectedness, and reduce health and social care costs (UK Government, 2019). Housing for older people is a UK Government priority and a taskforce has been established to further develop the sector (UK Government, 2023). Despite the developing market, there remains comparatively little extra care housing (ECH) relative to other forms of supported housing or care home (Housing Learning and Improvement Network (Housing LIN), 2019).

ECH is a model of housing with care and support designed to enable older people to age in place (Atkinson et al., 2021; Housing LIN, 2015). In contrast to residential or nursing care homes, residents have a private, self-contained flat or apartment, with a range of tenures available, access to various communal facilities, and 24-h onsite care and support provision. Residents can be private owners, leaseholders, rental tenants, or social housing tenants. It is also key to understand that care and housing costs remain separated, meaning the financial costs of care and support are also a mixed economy. A crucial feature of ECH is that care provision is flexible and adapts to permanent or temporary changes in need. Various terminology is used to describe ECH including very sheltered housing, housing with care, integrated care, retirement villages and assisted living (Barrett, 2023). All terms are used to indicate an age-exclusive setting that provides more services and facilities than traditional sheltered housing, where only a warden facility may exist (Barrett, 2023). There is an estimated 1980 ECH sites in the UK (Elderly Accommodation Council, 2021). There is variety in ECH provision across the UK including differences in location, size, facilities, and availability of specialist support (Atkinson et al., 2014). Approximately one fifth of residents in ECH are living with dementia (Barrett, 2020a) and yet, there remains gaps in knowledge with regards good practice supporting people with dementia in ECH (Atkinson et al., 2023; Barrett et al., 2016; Smith et al., 2022).

Previous studies have highlighted key benefits in ECH that can support residents living with dementia, including dementia-friendly design, promotion of independence, flexible care provision, appropriate use of technology, opportunity to live as a couple, a sense of safety and security, home ownership and social inclusion activities (Atkinson & Oatley, 2023; Atkinson et al., 2023; Barrett, 2015; Dutton, 2010; Evans et al., 2020; Twyford, 2016).

ECH can be grouped into three different models of provision (Barrett, 2012). Most ECH sites in the UK are integrated (Barrett, 2023; Twyford, 2016), offering accommodation for people living with dementia alongside residents without. Precise data on the prevalence of models is unavailable, although a recent survey found 89% of sites were integrated (Barrett, 2023). Specialist ECH offers accommodation that is only for people living with dementia, although non-cognitively impaired spouses may also choose to live there. Finally, separated models offer a separate area or unit for people living with dementia within a large integrated site. The prevalence of different models, and how different models of provision offer (or impede) potential benefits has remained under researched (Atkinson et al., 2023; Dutton, 2010; O’Malley & Croucher, 2005). Limited case study evidence suggests specialist models are usually smaller, have higher staff-to-resident ratios and can therefore offer more intensive support that is more effective at managing challenging symptoms (Barrett, 2012; Burns et al., 2009; Garwood, 2008b). This has led some authors to conclude that specialist models might be able to support people to live in place for longer (Barrett et al., 2016). However, other research has suggested specialist model provisions that better support people with dementia also limit independence and therefore undermine the ethos of ECH (Evans et al., 2020). In contrast to specialist models, evidence from integrated sites has suggested residents without dementia can offer some positive support to those living with the condition (Barrett, 2020b; Twyford, 2018). However, there also remains frequent reports of stigma and social exclusion of residents with dementia in integrated models (Evans et al., 2020; Evans & Vallely, 2007; Twyford, 2018; Vallely et al., 2006).

Limited awareness of ECH and a lack of evidence provides problems for professionals involved in adult social care assessment, care planning, and local authority-based adult social care commissioning (Smith et al., 2017). The primary role of such commissioners is to procure contracts with care providers in order to create a mixed economy of care suited to the needs of their local authority (Smith et al., 2017). A recent All-Party Parliamentary Group enquiry concluded that more research was needed to provide evidence-based information about housing choices available for people with dementia (Twyford & Porteus, 2021). In addition, research into the different models available for people living with dementia has been repeatedly called for (Atkinson et al., 2014; Atkinson et al., 2023; Barrett et al., 2016; Dutton, 2010; Twyford, 2016). This is a priority in adult social care research (Cyhlarova & Clark, 2019).

The overarching aim of the study on which this paper reports was to explore experiences of living with dementia in ECH and this has been reported elsewhere (Atkinson & Oatley, 2023). This paper presents data about the potential benefits and challenges of different models of provision for people living with dementia and those that care for them (professionals and family care partners).

## Methodology

The paper reports on findings from a large project funded by the NIHR School for Social Care Research (102645/ER/UWTA-P180), which explored how different models of ECH could support people living with dementia.

This paper considers the following research question:

What are the advantages and disadvantages of different models of ECH for people living with dementia and those that support them?

Data generation involved semi-structured qualitative interviews with 100 participants across eight case study sites. An interview schedule is provided in supplemental materials. Participants are detailed in Table 1. All interviews were face-to-face in a private room within each site. Interviews with adult social care professionals were undertaken online using Microsoft Teams.

**Table 1.** Participants by site and type with average interview duration.

	Resident with dementia	Resident spouse (without dementia)	Family member (non-resident)	Resident without dementia	Site staff^1^	Adult social care commissioners and social workers^2^
Site 1	3	1	2	2	4	1
Site 2	4	1	0	1	5	3
Site 3	3	3	0	2	5	1
Site 4	4	0	1	1	3	1
Site 5	6	0	2	0	4	3
Site 6	4	0	0	2	5	1
Site 7	5	1	0	0	4	2
Site 8	5	0	0	1	4	0
Total number	34	6	5	9	34	12
Average duration of interviews	00:28:17	00:45:56	00:36:25	00:45:56	00:30:46	00:34:14

^1^Staff included participants who were employed at the ECH sites in the study and included site managers, housing, administrative, and care staff.

^2^Adult social care professionals included local authority-based adult social care commissioners and social workers.

Case study sites were purposively selected to represent the three models under investigation (integrated, specialist, separated). Sites were identified through desktop research, the National Institute for Health and Care Research (NIHR) Enabling Research in Care Homes (ENRICH) network, and the project advisory group. The project advisory group was made up of professionals with expertise in housing, adult social care, dementia, and people with lived experience of dementia in ECH. The desktop search was undertaken between October 2021-March 2022 using the Elderly Accommodation Council ‘Housing Care’ website that lists retirement housing in the UK. Where the model was unclear, email/telephone contact was used to confirm. A sampling frame was created and sites were purposively recruited to include different sizes, locations, and facilities. Initial intentions had been to recruit three sites per model, but only a single separated site was identified (Site 7). A former separated site (Site 6) was also included as the transition was recent and several staff had worked since prior to the transition. Details of sites identified are provided in Table 2. All sites were run by not-for-profit organisations.

**Table 2.** Summary of case study sites.

Site	Location	Number of apartments	Facilities	Model
Site 1	Town	40	Shared lounge, restaurant, laundry, shop, hobby room, garden	Integrated
	Midlands			
Site 2	Town	42	Shared lounge, restaurant, laundry, hairdresser, garden	Specialist
	Midlands			
Site 3	Metropolitan area	49	Shared lounge, laundry, garden	Specialist
	Southeast England			
Site 4	Metropolitan area	260	Village hall, restaurant, gym, hairdresser, shop, hobby room	Integrated
	Midlands			
Site 5	City	33	Shared lounge, garden	Specialist
	Midlands			
Site 6	Rural town	40	Shared lounge, restaurant	(Former^1^) separated
	Northeast England			
Site 7	City	70 (50 integrated, 20 separated)	Shared lounge, restaurant, laundry, hairdresser, hobby room, garden	Separated
	Northeast England			
Site 8	Large village (population 14k)	54	Shared lounge, restaurant, laundry, hairdresser, hobby room, garden	Integrated
	Midlands			

^1^Site 6 had previously been separated with apartments in a specific area separated from the rest of the site by closed doors. This separated area had been integrated into the broader site when data generation in this study occurred. Specific dates are not provided to ensure anonymity of the site.

Ethical approval was granted through the Health Research Authority (21/HRA/3769). Participants living with dementia were identified by senior staff members at each site based on diagnosis recorded on their care plan. The process consent method (Dewing, 2007) was used to assess the capacity of residents living with dementia to consent to participation. The process consent model is a relationship-based model that ensures the researcher understands how the participant will communicate objection or withdrawal of consent (Dewing, 2007). This involves preparation work before any data generation occurs to understand the individual’s capacity, communication ability and wellbeing. As per the *Mental Capacity Act 2005,* all persons were deemed to have capacity to consent unless it could be demonstrated otherwise. Preparation work involved providing an information sheet and discussing it with the participant. The discussion established whether the participant could (1) understand the information, (2) retain, and (3) weigh up the information to (4) communicate their decision as per the *Mental Capacity Act 2005*. All participants interviewed had capacity and provided written consent. On reflection, this was likely a result of staff gatekeepers selecting only those residents they believed had capacity to participate. In the event a participant had not been able to provide consent, a personal consultee process had been approved by the ethics board.

All participants without dementia (e.g., residents, staff) were also identified by senior staff members at each site. This was based on an information sheet and discussion with researchers about the nature of the study. This included introductions to external adult social care professionals. At Site 8, despite multiple attempts, no response was received from a related adult social care professional. All participants were given information sheets and provided written consent. Following each site visit, the research team discussed the interviews undertaken. Interviews were audio-recorded and transcribed, removing any identifying information, and replacing names with pseudonyms.

The underpinning theoretical framework was informed by a contextual constructivist position that recognises multiple social reality interpretations are valid and dependent upon the context of the research (Madill et al., 2000). This position recognises language as the mode through which understanding about the world can be developed (Edley, 2001). Template analysis (Brooks et al., 2015; King, 1998) was used to identify recurring aspects of the data that would answer the research questions (Braun & Clarke, 2022). An initial template was developed following analysis of early interviews across participant types. Initial codes included both deductive codes based on a literature review (e.g. social inclusion; Atkinson et al., 2023) and inductive codes based on the data (e.g. peer support). Coding and analysis occurred simultaneously with further data collection. RO listened to all interviews and both authors coded the data. The research team met regularly to refine the coding template. Coding examples were also discussed and refined with the advisory group with group members providing feedback as to the credibility and quality of coding and interpretation. Data were coded across and between different participant types and models at both latent and semantic levels.

## Findings

Data demonstrated that there were advantages and disadvantages across different models for different stakeholders. Table 3 presents a summary of main advantages and disadvantages to residents living with dementia and staff in ECH by model type. Sources of quotations are made clear and [brackets] are used to clarify meaning or abbreviate quotations. Resident spouse is used to identify that the person was living in ECH with a partner with dementia.

**Table 3.** Summary of main advantages and disadvantages to residents living with dementia and staff in ECH by model type.

	Integrated	Specialist	Separated
Advantages	*Benefits to resident* Can be large and contain many onsite facilities providing lots of choice about where to spend time, what to do, and who with, without the need to leave the site.	*Benefits to resident* Often smaller and therefore, smaller area and smaller number of people for resident with dementia to become familiar with.	*Benefits to resident* Having a separated area can provide a smaller area and smaller number of people for a resident with dementia to become familiar with.
	Sense of ‘normalcy’ living alongside people without cognitive impairment	Couples where both parties are living with dementia can continue to live together.	Can be a large site that has many onsite facilities and activities, providing lots of choice about where to spend time, what to do, and who with, without the need to leave the site.
	Non-cognitively impaired residents can offer some informal care and support to residents with dementia.	Shared understanding of dementia between some residents living with dementia.	Residents can choose to access facilities and activities of broader site if willing and able to.
	Couples where one party is living with dementia can continue to live together with support as required.	Residents with milder symptoms could provide support to others, making use of their strengths and providing purpose.	*Benefit to staff and resident* The separated area usually has higher staff: resident ratios, providing more intensive support, which could prolong a person’s residence, as well as support staff to manage more challenging symptoms in a smaller area.
	Access to onsite social activity for a spousal carer without cognitive impairment	Staff trained to understand and support people living with dementia, therefore motivated to find ways to support symptoms that are more challenging.	
	Having specialist staff trained to support people and families living with dementia could ameliorate some of the disadvantages.	*Benefit to staff and resident* Models often have additional technological or physical barriers to make exiting the site more difficult. This can support some people to stay safe, as well as be advantageous to staff.	
Disadvantages	*Challenges to resident with dementia* Risks of experiencing stigma views or exclusionary behaviour from other residents	Smaller sites usually have fewer facilities onsite, therefore reduced choice to residents over how/where/with who to spend time.	*Challenges to staff and residents* Staff may be required to support transition into broader integrated area site for facilities/activities.
		Residents who lack insight into their condition might express stigmatising views or demonstrate exclusionary behaviour towards others.	
	Potential risks of unsupervised access through unlocked exits to the outside world.	Residents with milder symptoms could be frustrated by the lack of social opportunity with residents with more advanced symptoms.	Separated area can serve to highlight dementia and result in stigma from other residents, which can be upsetting/exclusionary to residents with dementia, and difficult for staff to manage.
	Maybe be insufficient staff: resident ratios, meaning insufficient care and support is available.	Staff time can be dominated by residents with more advanced symptoms, making it harder for residents with milder symptoms to access support.	
	Risks of loneliness in private flat without proactive staff support.		
	*Challenges to staff* Challenge to staff in managing the social dynamics of people with varied symptoms of dementia living alongside people without cognitive impairment.		
	Challenge to staff in managing people with more advanced symptoms (e.g. wanting to exit the building or becoming disoriented).		

### Models of provision

Most sites identified by the search strategy were integrated. Although the strategy was not exhaustive, this finding is supported by previous literature (Barrett, 2023; Twyford, 2016) and indicates that there is limited choice with respect to different models available.

### Integrated models

Being the most common, integrated sites offered the greatest choice in terms of location, size, tenure, and facilities. Professionals believed that integrated sites could offer a sense of normalcy that was beneficial to the daily routines of residents living with dementia:

“It would be nice to have a [mixture] because [residents with dementia] have a more normal life to follow” (Commissioner, Site 4)

A particular emphasis was on potential social interaction between residents living with and without dementia:

“We don’t like to just sit them altogether because they can't often communicate, so we try to sit them with somebody else that might start a conversation up” (Staff 1, Site 1)

Residents living with dementia also spoke of positive relationships with other residents and the valuable social opportunities their site offered:

“I can meet [other residents] or go to the lounge and all that. I chat to them. It’s a thing I have never experienced before you see […] As soon as I got out and met people, I loved it.” (Frank, resident with dementia, Site 8)

“We’ve got the best neighbours you could ever have next door and they take us out, don't they?” (Andrew, resident with dementia, Site 3)

However, both residents and staff recognised that managing the social dynamics of an integrated site could be challenging:

“When you have someone with dementia, and they express certain behaviours or appear different, that can be really challenging because we’ll have comments sometimes like, ‘I've bought my property and it’s not a nursing home’” (Staff 5, Site 3)

“It’s independent living, people want to see that people round them can live independently” (Betty, resident spouse, Site 3)

Some residents without dementia expressed concerns that their site provided insufficient support for those living with dementia:

“I don’t think that they can care for them enough. They wander about, they don’t know where they are” (Sara, resident, Site 1)

“If a family put somebody in here with dementia, they’re going to need a lot more care than can be given because it isn’t cheap [...] They’re in the wrong place” (Marie, resident, Site 3)

Such views were based on symptoms more commonly associated with advancing dementia and indeed, there was evidence of similar stigma towards other residents without dementia who had higher level care needs. However, there was also data that suggested residents could be nurturing and supportive of residents living with dementia:

“We knew Bonnie had got dementia, she lived over there [...] If we seen her, we'd keep an eye and things like that” (Gill, resident, Site 3)

“If I’m approached [for help]…there are a couple [of people with dementia], it doesn’t bother me, if it’s going to take me five, ten minutes out of my time, well, I’ve got plenty time” (Charles, resident, Site 8)

A particular challenge within integrated sites could be managing the unlocked front door. Whilst some residents living with dementia continued to access the outside world independently and had the capacity and desire to do so, the risks of doing so for others could be difficult for staff to manage.

For example, Site 3 had facilities that were open to the local public. Although there was fob-access to residential areas, front doors were automatic and there was little attention to people entering and leaving the site:

“There’s been instances where we’ve had to run after some residents to try, for their own safety really, and coax them back in” (Staff 3, Site 3)

In Site 1, there were similar challenges managing the front door:

“All you’ve got to do is press a button and the door opens. We try and persuade people not to go out if we don't think they [should], but we can't physically stop them” (Staff 1, Site 1)

An advantage of integrated sites was that they could offer accommodation for couples where only one party was living with dementia. This meant that couples could continue to live and care for each other with additional care and support as required. As dementia symptoms advanced, some couples made use of external day services to give the partner without dementia a break from their caring role:

“On a Monday and a Friday [my husband] normally goes to a day centre. So, I have those two days, which I can go down and have lunch with [other people]. We have lunch and then we have a coffee after that.” (Delia, resident spouse, Site 3)

This enabled spousal carers to access the social activities and peer support available in the wider site, which in turn, could support them to sustain their caring role for their partner. Having such support from a partner could enable a person living with dementia to remain living in place for longer (although more intensive support in residential care may still be required at some point). If the partner without dementia died first, this could prompt a rapid relocation under crisis if the partner with dementia required more support than staff could provide in ECH.

Staff reported certain symptoms of dementia to be more difficult to manage across integrated models. For example, walking with purpose (Barrett et al., 2020), aggressive behaviour, frequent disorientation, trespassing into private flats, and high levels of distress could trigger complaints from other residents. In turn, complaints added an additional challenge for staff to manage, alongside trying to find ways to reduce the risk of such symptoms. It was common for other residents, staff, and social care professionals to recognise that integrated models were insufficiently resourced to manage advancing symptoms of dementia:

“I just feel that they need more care. We can’t do it here.” (Sara, resident, Site 1)

“We’ve only got so many carers.” (Staff 3, Site 3)

For residents living alone with dementia, there was a particular risk that they could become increasingly confined to their flat, resulting in loneliness:

“I just, get fed up doing nothing really” (Jenny, resident with dementia, Site 1)

There was a risk that privacy could be isolating if you required additional support to access social opportunity:

“I would say it’s friendly, but you’ve got to join in. You need to go down and have a coffee, or…it’s no use just sitting in your room. Because people won’t come up to your room. Nobody interferes with you” (Delia, resident spouse, Site 3)

Two of the three integrated sites in this study had specialist staff who ran activity programmes and supported residents with dementia, and/or advised other staff on dementia. This was valued:

“I felt like I belonged somewhere, not to the [site], but to [programme]. The community that’s built within there” (Andrew, living with dementia, Site 3)

### Specialist models

Specialist models offer housing that is exclusively for people living with dementia, including providing for couples where both parties are living with dementia. Seemingly typical of specialist provision, sites in this study were smaller and had comparatively few communal facilities relative to integrated sites (Barrett, 2012; Burns et al., 2009; Garwood, 2008b).

Data suggested that specialist provision could create a sense of shared understanding between residents:

“Here, if someone says something silly, it's okay, because more other people say it. They might have more of an understanding and feel not on their own here” (Staff 3, Site 4)

“I’m not a forward person, and I’m not very good with my memory, but [staff and other residents] always understand what I’m saying” (Katharine, resident with dementia, Site 2)

Not all residents had insight into their condition and (just as in integrated models) there was evidence of stigma amongst residents:

“A couple of people think people [sundown] on purpose and of course they don’t and that can cause a bit of conflict” (Staff 3, Site 2)

However, there was also evidence of residents providing support for each other, making use of each other’s strengths:

“The girls are busy obviously, and [another resident is] asking me, ‘I need to go’, but she forgets [...] I buzz down for her, and [the staff] say, ‘alright, we’ll be up in a minute’” (Bryony, resident with dementia, Site 4)

Residents valued the activities and social opportunities that the site provided. Having staff in place to facilitate interaction was important:

‘The room downstairs, there’s always somebody there to see to anybody or do things or what’s going on’ (Annette, resident with dementia, Site 2)

Managing who was entering or exiting the site was more tightly controlled across the three specialist models in this study, in comparison to the integrated models. All front doors required fob-access and two of three had a further fob-controlled door between the entrance hallway and residential areas, providing extra barriers to negotiate to leave the facility. Although residents at Sites 2 and 5 were issued with fobs on arrival, many residents were unaware of the technology, had lost their fob, or family had taken it away, thereby limiting their freedom and potentially breaching their human rights. All but two residents at Site 3 were subject to Deprivation of Liberty Safeguards (DoLS) and did not have a fob. DoLS are part of the Mental Capacity Act 2005 that provide a legal framework in the UK to ensure people are not illegally deprived of their freedom. The varied use of DoLS across case study sites indicates local variety in interpretation and application, and warrants urgent further exploration and legal expertise.

Some residents who were living with milder symptoms of dementia in specialist sites expressed frustration at being surrounded by residents living with more advanced symptoms of dementia:

“Most of the people who live here are all ga-ga anyway. They’ve got dementia and stuff like that, and I just back off from that. Because I know that’s the way I’m going to be later on and it’s not nice” (Peter, resident with dementia, Site 5)

“[Other residents] have got no conversation, they don’t talk. You have to try and force them all the time” (Anne, resident with dementia, Site 5)

Such residents could find that staff time was dominated by those with higher support needs, undermining their own access to staff support. Some professionals suggested that specialist models were better suited to those with more advanced dementia:

“I think it depends what stage you’re at. Some of them, I think it’s too early for them to be in a place like this” (Staff 4, Site 5)

Yet, others recognised the mix of care needs was necessary to underpin the ethos of ECH:

“[For] a vibrant active community, you need those people without care and low-level needs in the building, otherwise you get the comments that it feels like a residential home” (Commissioner, Site 2)

Staff in specialist models described being accepting of challenging behaviour, or trying to find ways to reduce levels of distress:

[She] wanders [...] [Staff] either put her back to bed or have her sitting there […] One day she’s walking round, and the next day she’s sleeping, and then because she slept, she’ll walk round again. You can’t stop her from walking round (Staff 3, Site 5)

“There’ll be the odd hiccup we can resolve with a change of routines, like the lady locking the doors. That brings her so much peace and then she can settle. It might not have worked. We tried about five other things first and they didn’t work” (Staff 1, Site 2)

The designated specialist nature of the model meant that staff and organisations were committed to findings ways to support people living with dementia to maintain their residency:

“I think we’re just quite passionate about trying to achieve the best outcomes, if that means we have to put a little bit more in then that’s what we do” (Staff 1, Site 2)

That said, even specialist provision could not promise to be a ‘home for life’, and both staff and residents reported that other people had had to be moved on to residential care for similar reasons as those reported in integrated models.

### Separated models

Separated models were rare, with only a single site currently offering separated provision identified by the search strategy. The concept of segregation raised concerns for some professionals in the study:

“I’ve always been a real big advocate of not segregating people [...] Ultimately, why should we do that?” (Staff 5, Site 3)

“My worry would be segregating people and therefore labelling them” (Social worker, Site 1)

The lack of separated models may reflect trends towards inclusive living. Site 7 had 70 one and two-bedroomed flats. Twenty of these flats were separated behind a fob-controlled internal door that was left unlocked (but not open) during the weekdays. The separated area had a large activity room used by the whole facility and its own communal lounge.

Staff and residents in Site 7 described multiple benefits to their model of provision echoing those seen in both integrated and specialist provision. As per other sites, residents spoke of the benefits of social activity and staff support. Any person living with dementia could move into a vacant flat in the separated area, but there were also people living with dementia across the rest of the site. Those residents were living with their partner, or developed symptoms of dementia after moving in. For the latter group, it was usually not in their best interest to relocate once within the site, although the manager from Site 6 (a site formerly offering separated provision) recalled this happening and it being a success in prolonging someone’s residence:

“If you had somebody living within the [site] whose dementia really deteriorated, [...] then rather than have to move them into residential or nursing care, when an apartment comes up... We had two or three that transferred down, kept them living longer [...] Families fought for that (Staff 3, Site 6)

Having a smaller separated area could be beneficial to residents living with dementia as they had fewer people and a smaller space to familiarize themselves with:

“There’s always somebody round about [...] Now they’ve got to know us, so much, which is nice” (Molly, resident with dementia, Site 7)

For staff, a benefit was the ability to provide more intensive support in a smaller area to people with more challenging symptoms of dementia. This was particularly in reference to managing ‘sundowning’ and walking with purpose:

“That’s why we have the lock on the door. Because some of them sometimes in the afternoon wander and get a bit confused” (Staff 2, Site 7)

“In here, there was only six flats, you could keep an eye on [people]” (Staff 3, Site 6)

This was suggested to be beneficial to all parties including those who were not living with dementia in the wider site:

“If you live in flat 42 and then you have a lady with dementia who’s really sort of progressing, [...] I just think it can be very unsettling for other people as well” (Staff 3, Site 7)

The previous separated area in Site 6 was suggested to have enabled people to live in place for longer:

“It’s just a shame for those people that, maybe, could have stayed in their own home for longer have had to move”. (Staff 2, Site 6)

In both sites, the fob-access internal door created an additional barrier between residents living with dementia and the broader site (and thus, the automatic opening front door), reducing the risk that people might leave the site without support if unsafe to do so. Residents in this area were not necessarily confined by the internal door. For those residents able, they could use a fob to access the wider site and broader community. This was invaluable to Richard, who (as per residents with milder symptoms in specialist ECH) preferred spending time with people unaffected by dementia:

“The people in [separated area], mostly are further on than I am with dementia. The people in [integrated area] are not. So, I’m able to converse with them better.” (Richard, living with dementia, Site 7)

For residents less able to manage a fob-accessed door, staff could support transition between different areas to access activities or the restaurant. However, there remain questions with respect to the management of a resident’s right to liberty in circumstances where they are unable to use the technology. Indeed, the manager at Site 6 reported their separated model was ended due to concerns about liberty deprivation.

The primary negative aspect of having a separated area reported in the data was that it could serve as a point at which stigma could be directed. Staff found that some residents could be intolerant of those with dementia being outside of their “area”:

“Some of [integrated area] they think it’s them and us. For example, a few Saturdays ago one of the residents from [integrated area] said to one of the staff members, ‘take them down there where they belong” (Staff 4, Site 7)

It should be noted that no resident living in the separated area who was interviewed here reported stigma. However, this absence of evidence should not be interpreted as absence of existence.

## Discussion

Findings in this study have demonstrated that there are potential advantages and disadvantages to different models of ECH provision for people living with dementia and those that support them. There is no model that can be universally agreed as the optimal choice for a person living with dementia. Rather, the findings reiterate the conclusion that ECH provision for residents with dementia is complex and must be person-centred (Brooker et al., 2011; Evans et al., 2020; Smith et al., 2022).

Integrated models offer a range of benefits including more choice (e.g. location, facilities, size), a sense of normalcy (so far as age-exclusive housing can provide), and social support and interaction. However, they also present challenges in terms of managing social dynamics, balancing safety and risk, dealing with dementia-related stigma, and providing sufficient specialist resource to meet the holistic needs of a resident living with dementia. Previous research in six integrated sites reported that residents with cognitive impairment were less socially integrated and reported some loneliness (Evans & Vallely, 2007). Findings here suggest staff could have an important role in facilitating social opportunity through reminding residents of planned activity or supporting with access. Reports of stigma in integrated models are common (Evans et al., 2020; Evans & Vallely, 2007; Twyford, 2018). Findings in this study replicate similar themes and yet, there was also evidence of residents without cognitive impairment offering support and understanding to residents with dementia. Similar evidence has also been reported recently (Barrett, 2020b; Twyford, 2018). Finding ways to reduce stigma and promote tolerance and support go beyond ECH provision to society in general, where reports of stigma remain common (Alzheimer’s Society, 2019). It is worth noting that stigma was present across all models in this study and seeking specialist services does not eradicate the risk. Stigmatised views can impact behaviour and result in the exclusion of people living with dementia, negatively impacting psychosocial health and dementia symptomology (Batsch & Mittelman, 2015; Kim et al., 2019). In this study, stigmatised views were rooted in dementia stereotypes of incapability and dependence, irrespective of individual’s actual support needs, suggesting that some residents believed no amount of support in ECH could enable a person with dementia to live in place.

Previous studies have suggested people living with dementia do better in ECH when they move in an earlier stage (Vallelly et al., 2006; Verbeek et al., 2019). This is explained by the retained ability to build new routines with new people at earlier stages of the disease. However, it is possible that the impact of stigma also affects the ability of people at different stages to live well in ECH. Regardless of model, achieving a balance of care needs (low, medium, high) is reported to be important with respect to matching demands with available staff resource (Batty et al., 2017; Darton & Callaghan, 2009; Wright et al., 2010). Thus, the diversity inherent within an ECH population is an aspect of concept design that must be made clear to potential new residents before arrival regardless of model of provision.

As per other case studies reported, specialist sites in this study were smaller and had fewer communal facilities (Barrett, 2012; Burns et al., 2009; Garwood, 2008b). In a previous study that included three integrated sites, and a specialist site, the lack of facilities in the smaller specialist site was interpreted to inhibit independence and reduce social opportunity (Evans et al., 2020). However, this was not reported by residents or staff in this study, although the importance of an activity coordinator to facilitate activity was noted. Although fewer facilities, the smaller location might be preferrable for some people living with dementia, who can more easily familiarise themselves with fewer people and a smaller place. Furthermore, the specialist knowledge and commitment of specialist models could support people to live in place for longer as has been previously suggested (Barrett et al., 2016). However, this does not demonstrate that separation is beneficial, so much as the approach to care is different in this context. Indeed, benefits of separate support (whether specialist or separated) could be interpreted as beneficial to staff rather than residents. For example, being able to confine residents who might be walking with purpose to a smaller area enables staff to more easily monitor and intervene and reduce the chance of complaints from other residents. As per this research, it has not been uncommon for integration to be reported as unpopular with residents without cognitive impairment (Evans et al., 2020; O’Malley & Croucher, 2005). Research into specialist care environments beyond the UK is mixed (Calkins, 2018; Zimmerman et al., 2022). Evidence from the USA suggests that the practice and dynamics within the environment are more important than the model of provision itself (Zimmerman et al., 2022). This aligns with the conclusion here that variables beyond the model must be person-centred to achieve the optimal individual environment. Whilst the politics of specialist care home provision have been described as unethical by some (Steele et al., 2019), and the evidence mixed (Steele et al., 2019), the reality is that few people have the choice of such a model of ECH given the lack of specialist or separated provision apparently available across the UK. Given the suggestion in this study that that specialist provision might support people to live in place for longer because of the dedicated nature of the service (to support people with dementia), a lack of specialist provision available in certain locations seems unfair.

The diversity of ECH in the UK makes conclusions with respect to models challenging and the reality is that different models will suit different people. Whilst smaller sites may be easier to navigate, larger sites offer more choice of activity, facility, and people to spend time with. Irrespective of model, specialist staff to support residents with dementia are not essential to ECH provision, but evidence here suggests that their role can be crucial to living as well as possible, echoing previous research (Barrett, 2020b; Brooker et al., 2011). That said, irrespective of the model, ECH cannot be a ‘home for life’ for residents living with dementia. Findings from this study echo previous research that recognised advancing dementia can result in a person being denied entry or encouraged to move on from ECH (Barrett, 2012, 2020b; Barrett et al., 2016; Croucher et al., 2007; Evans & Vallely, 2007; Garwood, 2008a, 2008b; Verbeek et al., 2019).

There are many potential benefits to living in ECH. However, there are also potential challenges and variables beyond the model of provision that affect the lived experience. Stigma, loneliness, advanced symptoms of dementia, lack of staff resources, inter-resident dynamics were potential issues across all models that could affect the lived experience of a resident with dementia.

## Conclusion

There is no universal model of best fit with respect to ECH for people living with dementia. Different models will work for different people. The key is that people have choices with respect to where they live. This includes a choice over specialist provision that might be able to support them to live in place for longer. Integrated models might be the ideological ideal, but aspects of such a model can be disabling. For example, the size of site, open door policy and lack of understanding of dementia from other residents and (potentially) staff could undermine residence within such a model. That is not to say that such issues of size/ability to navigate, unsupervised/open exit doors, and a lack of understanding of dementia might also occur in specialist models of provision, but perhaps staff at such models are more inclined to find ways to sustain a person’s residence given the specialist nature of their mission. Developing ECH requires ongoing investment and promotion of ECH as a viable option for older people and people living with dementia. Future research must consider cost-benefit analysis of the different models (this was not included in this research) and utilise longitudinal approaches to understand how different models work as symptoms progress across time. The latter will provide further insight into the lived experience, whilst the former is important information that will affect future local authority commissioning decisions, as well as the decision for a privately funded potential resident to access ECH or another form of care.

## Limitations

The sample included was purposive and intended to include diversity of location, size, and facilities (as well as model). Future research to provide a detailed analysis of ECH stock in the UK would provide further insight into how representative this sample was. However, there are no claims of generalisability given the qualitative nature of this study. The focus was on gathering rich data about lived experiences, to provide insight into a significant gap in research.

The population sample in this research was entirely White British (although efforts had been made to prevent this by targeting areas with greater ethnic diversity). Limited ethnic diversity is common in ECH (Darton et al., 2012) and findings here suggest this still could be the case. There remain significant gaps in knowledge with respect to the experiences of ethnic minority and LGBTQ + residents living in ECH, and future research would also do well to explore what factors and practice can affect diverse lived experiences within the context. In addition, exploring the barriers and facilitators to relocating to ECH for a diverse range of people living with dementia would be of value in developing the potential of ECH in addressing increasing health and social care costs, as well as the shortfall in appropriate housing for older people in the UK (UK Government, 2015, 2019). It should be noted that this was not a comparative study. People usually only have experience of living in one type of ECH and such is the diversity of sites even within a model type (e.g., integrated sites can vary in size, location, and facilities), it is challenging to make overarching comparisons by broad model type.

A further limitation is recognition that this study did not record the total number of people living with dementia at each site, nor did it assess the stage of dementia for those living there. Given that the sampling included only those that had consent capacity, any residents with later-stage dementia were perhaps (unintentionally) excluded from interview. As suggested above, this was likely a result of gatekeeping by staff involved in identifying potential participants, but does add an additional potential limitation and avenue for future research that specifically considers those with later stage dementia.

That said, by exploring the lived experiences of residents, staff and external professionals, this paper has provided new insight into the relative advantages and disadvantages of different models of ECH for people living with dementia and those that support them. This provides additional awareness of factors for people living with dementia and those that support them when considering the design, management, resourcing, relocation to ECH, or support of a relative within ECH.

## Supplemental Material

Supplemental Material - A qualitative study of the benefits and challenges of different models of extra care housing for residents living with dementia Supplemental Material for A qualitative study of the benefits and challenges of different models of extra care housing for residents living with dementia by Rebecca Oatley and Teresa Atkinson in Dementia

## Data Availability

The data that support the findings of this study are available from the corresponding author, [TA], upon reasonable request.
