# Supplementary material for: A qualitative study of the benefits and challenges of different models of extra care housing for residents living with dementia
Source: Dementia (London). 2024 May 3;23(6):981–1000. doi: 10.1177/14713012241249794 (PMC11290033; doi:10.1177/14713012241249794)
Supplement: Supplemental Material - A qualitative study of the benefits and challenges of different models of extra care housing for residents living with dementia [file sj-pdf-1-dem-10.1177_14713012241249794.pdf]

Appendix 1. Interview schedules for semi-structured interviews

The following interview schedules acted as a guide to the topics covered in interviews.

Questions were tailored specifically to the individual participant (communication preferences, strengths, and support needs) and the type of ECH that they live in, as well allowing the interviewee to direct the nature of the conversation to topics most relevant to them.

Schedule 1: Residents living with dementia

Schedule 2: Residents without dementia

Schedule 3: ECH staff

Schedule 4: Adult social care commissioner

Interview Schedule 1: Residents living with dementia and/or family members

Question topics

Advantages/Disadvantages – independence/support balance, design (internal/external), facilities, social opportunities, relationships with staff/family/other residents, access beyond ECH

The site:
- Decision to move
- Understanding of extra care housing, independent living
- Facilities (what facilities are available, what do you use, what is good about the facilities, is there anything that is not so good/are there any facilities you don't have that you would like)?
- Flat (what do you like/dislike/prefer, do you have/use any technology)?
- Activities (what activities happen, who organizes them, what do you enjoy, how do you choose what activities you do, how/when/why do you join in activities, anything that stops you joining activities?)

The tenancy:
- Tenure, management

Activities of daily living:
- Daily routine, meals, shopping, hobbies/activities, other people
- Do you have/use any technology (e.g. ipad, internet, assistive technology)?
- Care and support plan – do you have one, what is included/not included, what works well, what doesn't work so well, what happens if you want more/less support?

Physical environment:

- Likes/dislikes

- Ease of navigation and signage
- Garden, restaurant, and other facilities

Social environment:

- Relationships (staff, family carers, friends, spouse, other residents, other facility users) within and outside of the site
- Access to activities off site?

Wider community:

- Is this your 'local' community or did you move into the area?
- Where/how do you access outside site?
- What do you like/dislike about the location?
- How do you access healthcare/off-site amenities (e.g. shops, hairdresser, podiatrist) when you need it?

Advantages/disadvantages:

- What do you think are the good things about living at [your site]?
- Is there anything that you would change about living at [your site]?

Interview Schedule 2: Residents without dementia

Question topics

- how long have you lived here, why did you choose to live here, is it what you expected?
- What do you like/dislike about ECH? Would you recommend to other people and why
- facilities (what facilities are there, what do you use, what is good about the facilities, is there anything that is not so good)
- activities (what activities, which do you attend, what is good, anything not so good, any changes you would make)
- relationships with other residents and staff – what is good/not so good, anything you would change
- do you think ECH is suitable for people living with dementia, are there any advantages or challenges for residents with dementia?

Interview Schedule 3: Extra Care Housing staff

Questions topics

- Experience working at [name of site]
- Understand ECH is? Differences from residential/nursing care?
- Who do you think this site works well for? Who do you think ECH works well for?
- Eligibility criteria for residents living with dementia?
- Move in/out procedures/transition support?
- Why do you think your site works well/not so well for residents living with dementia?
- Advantages of your model over someone with dementia living in their own home/other institutional care/other ECH model?
- Barriers or facilitators to you supporting a resident living with dementia here?
- Barriers or facilitators to the person living well here?
- Staff/resident relations with/without dementia?
- Any specialist dementia provision/staff/training/ activities at your site?
- Is there a point where ECH doesn't work so well?
- Benefits/challenges of integrated/specialist/separated models?

Interview Schedule 4: External adults social care professionals

Question topics

- Understanding/experiences of ECH?
- How does ECH fit with other types of accommodation and why?
- Who are residents who benefit the most?
- How does ECH work for people living with dementia?
- What models of ECH do you commission? How do you decide which models to commission?
- Key challenges/key advantages in ECH models (integrated/specialist/separated)?
- Barriers/facilitators to people living well with dementia in ECH?
- Barriers/facilitators to staff supporting residents with dementia?
- Advantages/challenges to the council with respect to housing people living with dementia in ECH?
- Outcomes for people with dementia in ECH
